# 

**Published:** 1809-12

**Authors:** 


### CORREPONDENCE.

We should not have required Juvenis's real signature had it not been from a delicacy in being obliged to quote the writings of one of the Editors.

Mr. J. M. C. will find the enquiry to which he alludes. The last edition of Dr. Adams's "Observations on Morbid Poisons," contains the following paragraph: "It has been said that the experiment made by Mr. Hunter has been since repeated with a different result. Can we wonder at this, when we consider from how many causes gonorrhœa may arise, and how impossble it is to distinguish the venereal from any other?"

We have, in another part of this Number, offered our apology for not inserting the Communications of Verax, and our learned Nottinghamshire Correspondent. We regret the necessity of refusing two pieces of real merit; but we conceive every practical information of too much importance to be discouraged, unless it is proved that we have been deceived. It is no disparagement to cobweb that it is an old remedy; and that there are instances in which it has failed of success. It has probably been at one time overvalued, like many other remedies; and, like them, been too suddenly consigned to oblivion. Like them, it must find its proper level; but it cannot be fair to admit all wit on one side, when nothing but matter of fact is offered on the other.

---

END OF VOL. XXII.

### INDEX.

ABERNETHY's, Mr. cafe of malformation of the heart 71
Abftinence, obfervations on 110
——— remarkable cafe of 337
Acetate of lead, fuccefsful exhibition of 350
Acid obtained from ginger 349
Adams, Dr. on carcinoma 80
——— on the leprofy 407
Æther, on fulphuric 362
Ague, benefits of cobweb in 96, 369, 371, 457
Air, experiments on atmofpheric 48
Alder, Dr. on the proximate caufes of difeafes 36, 296
——— proofs of the theory of 227, 458
Aldis's, Mr. cafe of enlargement of the heart 513
Alexandre's, M. method of filtrating water 139
Alkalies, on the decompofition of the fixed 11
Allen's, Mr. experiments on atmofpheric air 48
Amaurofis, cafe of 353
Amenorrhœa, obfervations on 46
Ammoniacum, account of the gum 360
Anatomy, on the ftudy of 3
Aneurifm of the carotid artery, cafes of 67, 69
Anguftura of commerce, account of the 144
Animal fecretions, on the 174
Anime, on the ufes of gum 360
Antimony pernicious in fever 41
Aphonia, cafe of 252
Arctopis, good effects of the 261
Armftrong's, Mr. cafe of purulent ophthalmia 364
Arfenic, new teft for detecting 87
———, in intermittents, on the ufe of 219
———, on the ufe of 337
———, cafe of death produced by 427
Afthma, cafe of 83
———, relieved by cobweb 375
Afthenia, cafe of 250
Atmofphere, on the influence of the 15
——— on the conftitution of the 177
——— on the natural hiftory of the 16

Babington's, Dr. cafe of the noxious effluvia of charcoal 152
Barbary, account of the plague in 193
Barclay, Dr. on the mufcular motions of the human body 5
Bartholomew's, St. hofpital, lectures at 350
Becquet, M. on the vibration of the iris 468
Beet-root a fubftitute for coffee 525
Bellot's, Mr. cafe of ftrictured inteftine 392
Bichat, on certain opinions of 253, 333
Bleeding in fever, on 459
Blizard's, Mr. cafe of intus fufceptio 158
Blood, on the gelatine of the 74
Bocker, Dr. on a cutaneous difeafe 432
Body, on the mufcular motions of the human 5
BOOKS, CRITICAL ANALYSIS OF NEW MEDICAL. ——— Medico-Chirurgical Tranfactions, by the Medical and Chirurgical Society of London, Vol. I. 65, 152, 240. Dr. Lambe, on Schirrhous Tumours and Cancerous Ulcers, 79. A Treatife on Local Inflammation, particularly applied to Difeafes of the Eye, by J. B. Serny, M. D. 167. The Edinburgh Journal, Nos. XIX and XX, 245, 333, 427, 513. A Practical Materia Medica, 342. A plain Statement on the comparative Advantage of the Cow-pox and Small-pox Inoculations, by T. Smith, M. D. 343. A Treatife on Hydrophobia, by W. Maryan, 343. Analyfis of the Carbonated Chalybeate difcovered near Stow, by R. Farmer, 346. Obfervations on the Hepatitis of India, by W. Saunders, M. D. 415. Examination of Prejudices entertained againft Mercury in Hepatic Complaints, by J. Curry, M. D. 415. Account of a Pfeudo Syphilitic Cutaneous Difeafe, by H. Bocker, M. D. 432. Obfervations on fome Difeafes of the Heart, by A. Burns, 505. Obfervations on Strictures of the Urethra, 517.
BOOKS, NEW MEDICAL, ANNOUNCED. Dr. Roberton's Treatife on Difeafes of the generative Syftem, 83. Mr. Wilfon's Pharmacopœia Chirurgica, 176. Mr. Ward on the external Ufe of Opium, *ib.* Dr. Saunders on the Liver, 264. Dr. Adams's Edition of Mr. J. Hunter, 528. Dr Clarke's Pharmacopœiarum, *ib.* Dr. Stancliffe's Chemical Experiments, *ib.* Dr Stokes's Botanical Materia Medica, *ib.*

Bostock, Dr. on the gelatine of the blood 74

Botany, observations on 108

Boucher, M. on an organic disease of the heart 137

Bougies, observations on 287

Boulay, M. on sulphuric æther 362

Bowel complaint, case of 434

Brain, case of tumour in the 161

Brande's, Mr. experiments on calculi 49

Bread, on the making of 403

Breast, case of cancer in the 355

Bristol, deaths by consumption at 239

Bubon galbanum, description of 33

Cahguala, description of 87

Calculi, experiments on 48

Campbell's, Dr. case of hæmatemesis 180

Cancer, observations on, with cases 80

Cancerous hydatids, case of 355

Carbonic acid gas, experiments on 48

Carditis, two cases of 252

Carmichael, defence of Mr. 481

Carotid artery, aneurism of the, 67, 69

Castleman, Mr. remarkable case of 344

Cataract, on the 475

Catarrh, observations on 347

Cayenne pepper, its effects in amaurosis 353

Cazal's, Mr. case of intermitting fever 496

Chalk stones, observations on 155

Chalybeate, analysis of a carbonated 346

Champignons, on the effects of 503

Chamseru's, M. cases of plica polonica 500

Charcoal, fatal effects of burning 152

Chelsea, account of an ophthalmia prevalent at 260

Cheltenham, analysis of the waters of 15

Chemistry, observations on 11

Chevalier's, Mr. cases of sudden death 158

Chlorosis, remarks on 46

Cholera, prevalence of 438

Chorea, case of 245

Clarke's, Dr. medical report for Nottingham 244

Cobweb, on the virtues of 94, 218, 369, 371, 457

——— relieves a constitutional asthma 375

Coffee, a substitute for 525

Cold, salutary effects of topical 233

——— applied to the scrotum 327

——— bath, uses of the 230

Conception, cases of false 245

Consumption, proportion of deaths from 239

Contagion, observations on 105, 513

Cooper's, Mr. A. cases of carotid aneurism 67, 69

——— S. case of hæmorrhage 341

Cope's, Mr. case of hæmorrhoids 488

Corunna, case of the sick returned from 428

Copal, fine varnish made from 351

Cordus valerius, account of 270

Correspondents, to, 88, 176, 264, 352, 440, 528

Corrosive sublimate, its efficacy 515

Cough, case of violent 70

Cow-pox inoculation, advantage of 343

Cretinism, account of 9

Crowfoot's, Mr. cases of carditis 252

Cupping, on the operation of 204, 376 380, 486

Curry, Dr. on hepatic complaints 421

Cutaneous diseases, on 109

Cynanche tonsillaris, observations on 257

Davy, Mr. account of the discoveries of 12

Deafness, instances of the cure of 191

Death, on transudation after 32

———, cases of sudden 158

Delorme's, M. cases of closing up the glottis 140

Diabetes, on the treatment of 110, 252

Dickson, Dr. on the nature of fever 99

Digitalis, on the effects of 93, 124

Diplomas, observations on medical 492

Diseases, proofs of the proximate causes of 36, 296, 458

——— on embarrasing 47

——— at Edinburgh, reports of 84, 169, 257, 346, 433, 522

——— in London, 86, 172, 259, 349, 437, 524

——— of seamen, observations on 373

Dispensatories, historical account of 265

Dogs, on the distemper in 240

Dundas's, Mr. case of diseases of the heart 73

Dysentery, acetate of lead recommended in 350

Edinburgh, report of diseases at 84, 169, 257, 346, 433, 522

——— Journal, review of 244, 333, 427, 513

Education, observations on medical 302

Edwards, Mr. on venereal virus 312

Electricity, analogy of Galvanism and 314

Emetics in fever, on 463

Epidemics, on 105

Errata 352, 440

Ether, sulphuric, process for preparing 174

———, observations on 362

Etna, account of a recent eruption of mount 175

Ewell, Dr. on acetate of lead 350

Experiments respecting atmospheric air 48

——— on calculi 49

——— on arsenic 87

——— on cahguala ib.

——— on upas 92

Experiments on the animal fecretions 174
——— galvanic 315
——— on ginger 349
——— on fulphuric ether 362
——— for filtrating water 439
Eye, obfervations on difeafes of the 167
——, effects of galvanifm on the 320

Fafting woman, particulars of the 337
Fauberge, M. on mercury and fcrofula 494
Fawell's, Mr. cafe of fuperfœtation 47
——— obfervations on 293
Fever, on the nature of 99
——, cafe of intermitting 496
Fevers, on the proximate caufes of 36, 296, 458
Fœtus, a new inftrument for extracting the (*with a plate*) 103
——— found in the abdomen of a boy 166
Founier, M. on determining the quantity of fpirit 351
Fofter's, Mr. cafe of lithotomy 155
Froft-bitten fcrotum, cafe of 220

Galvanic influence, on the 313
Gas, experiments on oxygen 48
Gaftrodynia, on the word 264
Gelatine of the blood, on the 74
Gerrard's, Dr. cafe of amaurofis 353
Ginger, an acid obtained from 319
Gland, on removing a fchirrhous parotid 221
Glafgow, lectures in the univerfity of 261
Glottis, cafes of a complete clofing of the 140
Gonorrhœal virus, on the 312, 391
Gorget, on improving the 207
———, on the cutting 224, 339
Gouty concretions, on 155
Grainger's, Mr. cafe of abftinence 337
Grapes, method of preferving 440
Gums, natural hiftory of the 33, 358
Guaiacum, obfervations on 360
Guy's Hofpital, remarkable cafe in 350
———, lectures at 262
Guy's, Mr. inftrument for extracting the fœtus 103

Hæmatemefis, cafe of 180
Harrifon, Dr. on medical reform 330
Harveian oration, account of the 438
Hearing, on the reftoration of 191
Heart, cafe of malformation of the 71
——, account of a peculiar difeafe of the 73
——, account of an organic difeafe of the 137
——, cafe of enlargement of the 513
——, Mr. Burns, on difeafes of the 505
Heberden's, Dr. Harveian oration 458
Hæmorrhage from the urethra, cafe of 341
Hæmorrhoids, favourable termination of the 488
Hepatic complaints, on mercury in 415
——— function, on the 421
Hepatitis of India, on the 415
Hernia, two cafes of ftrangulated fcrotal 233
Hill, Mr. on the effects of arfenic 337
Home, Mr. on the functions of the ftomach 5
——— on ftricture, animadverted on 17, 126
Hooper's, Mr. account of difeafes at Plymouth 428
Hooping cough, on the treatment of 71, 173
———, query concerning 260
Hoffack, Dr. on contagion 513
Howfhip's, Mr. cafes of lock-jaw and tetanus 120, 185, 324, 396, 479
Hume's, Mr. method of detecting arfenic 87
Hunold, Dr. on perforating the tympanum 311
Hydatids, cafe of 140
——— in the breaft, cafe of cancerous 355
Hydrocele cured with port wine and water 205
Hydrocephalus internus, on 258
———, remarks on 378
Hydrophobia, Dr. Valentin's remarks on 53
———, Mr. Royfton on 113
———, Dr. Marcet's cafe of 157
———, Dr. Jenner on 240
———, Mr. Oldknow's cafe of 251
———, propofed differtation on 260
———, Mr Maryan on 243
Hydrothorax, on a cafe of 29
Hyfteric affections, cafes of 171

India, on the hepatitis of 415

Inflammation, treatife on local 167

Influenza at Edinburgh 171

Intelligence, medical, &c. 87, 173, 260, 349, 440, 525

Intermittents, obfervations on 189

Inteftines, on an injury of the 150

———, cafe of ftrictured 392

Inftrument for extracting the fœtus, defcription of an 103

Intus-fufceptio, cafe of 158

Iris, on the vibration of the 468

Iron, violent cough cured by preparation of 70

——, ufe of the pyrolignite of 439

Jackfonian prize queftion for 1810 260

Jackfon's, Mr. defcription of the plague 193

Jackson's, Dr. remarks on cobweb 369

Jarrold's, Dr. anthropologia, account of 8

Jenkinson, Mr on phthisis pulmonalis 89

——— on digitalis 93

——— on arsenic 337

Jenner, Dr. on the distemper in dogs 240

———, his two cases of small-pox infection 242

Jevans's, Mr. case of spontaneous recovery 2[illegible]7

Jones's, Dr. case of aphonia 252

Kinglake's, Dr. cases of strangulated scrotal hernia 233

——— case of hæmorrhage 327

Kino, description of the gum 361

Knee joint, cases of enlargement of the 515

Knives, case of a man who swallowed 350

Lambe, Dr. on cancers, &c. 79

Lancet for operating in ophthalmia described 42

Laudanum, effects of taking a quantity of 78

Lead, successful exhibition of the acetate of 350

Leath's, Mr. new lancet for the ophthalmia 42

Lectures, medical announced.— Dr. Clough, on Puerperal Anatomy, 87, 263, 528. Dr. Squire, on Midwifery, 88. Dr. Ramsbotham, on ditto, 176. At the University of Glasgow, 261. At St. Thomas and Guy's Hospitals, 262. At the London Hospital *ib.* Dr. Anderson, on Chemistry and Pharmacy, *ib.* Dr. Buxton, on the Theory and Practice of Medicine, *ib.* Mr. Carpue, on Anatomy and Surgery, *ib.* Dr. and Mr. Clarke, on Midwifery, 263. Dr. Clutterbuck, on the Theory and Practice of Physic, &c. *ib.* Dr. Hooper, on the same, *ib.* Dr. Reid, on the Theory and Practice of Medicine, *ib.* Mr. Robertson, on Midwifery, *ib.* Dr. Squire, on the same, 264. Mr. Brookes, on Anatomy, Chemistry, and Surgery, *ib.* Mr. Taunton, on Anatomy, &c. *ib.* Mr. Thomas, on Surgery, *ib.* Mr. Wilson, on Anatomy, &c. *ib.* At St. Bartholomew's Hospital, 351. Mr. Carlisle, on Surgery, 352. Dr. Satterly, on Clinical Instruction, *ib.* Mr. Moor, on the Teeth, *ib.* Dr. Ramsbotham on Midwifery, 528.

Leprosy, historical account of 407

Lincolnshire Medical Society, resolutions of the 323

———, remarks on the 465

Liquids, apparatus for determining the quantity of spirit in 351

Lithotomy, case of 155

———, improvement in the gorget for 207

Lock-jaw, cases of 120, 324

London, reports of diseases in 86, 172, 259, 349, 437, 524

———, on medical education in 302

——— Hospital, lectures at the 262

Lunn, Mr. on removing a schirrhous parotid gland 221

Luxmoore, Mr. on strictures of the urethra 517

Lyall, Mr. on cupping 388

Majendie, Dr. on the effects of vegetables on the spinal marrow 96

Man, on the natural history of 8

Manna extracted from rhododendron ponticum 261

Marcet's, Dr. case of hydrophobia 157

Marrow, effect of upas on the spinal 96

Maryan, Mr. on hydrophobia 343

Medical practice, on difficulties in 45

——— education, hints on 302

——— reform, resolutions respecting a 323

——— observations, on 465

Medicine, sketch of the progress of 1, 105

Medusa, description of the 526

Melæna, case of hæmatemesis with 180

Mercury, unsuccessful in tetanus 185

——— in hepatic complaints, on 415

———, on the use of in fever 462

Mercury and scrofula, observations on 494

Mind, importance of pleasing it in diseases 463

Mitchill, Dr. on the atmosphere 177

Moore, Mr. on gouty concretions 155

Moffman, Dr. answer to 124

Nacquart, Mr. on an injury of the intestines 150

Natural history of the gums 33

Navy, prejudices in the 29

——— surgeon, on the duties of a 374

Nervous influence, on the propagation of 161

——— disorders, observations on 208

Nose, remedy for bleeding of the 327

Nottingham, medical report for 244

Oldknow's, Mr. case of hydrophobia 251

Ophthalmia, new lancet for operation in 42

——— at Edinburgh, account of the 170

———, case of purulent 252

——— at Chelsea 260

———, Mr. Armstrong on 364

Ophthalmia, Mr. Simmons on 457

——, obfervations on 523

Opium, new method of preparing the extract of 351

Organic difeafe of the heart, account of an 137

—— injury of the inteftines 150

Oxygen gas, experiments on 48

—— in germination, account of 109

Parmentier, M. on opium 351
Parotid gland, on removing a fchirrhous 221
Patrick, Mr. on nervous diforders 208
Pearfon, Dr. on hooping cough 71
Pepys, Mr. on atmofpheric air 48
Pharmacopœias, hiftorical account of 265
Phthifis pulmonalis, on reft in 89
——, mortality from 239
Phyfician, port-folio of a 403
Phyficians, anniverfary meeting of the college of 438
Phyfiology, on the ftudy of 3
Pitcairn, Dr. on the cafe of 176
Plague, account of a peculiar fpecies of 193
Planche's, Mr. account of anguftura 144
Plants, on the germination of 109
Plica polonica, cafe of 500
Plymouth, ftate of mortality from phthifis at 239
——, difeafes of foldiers landed at 428
Pneumonia, cafes of 84
Port-folio of a phyfician 403
Practice, difficulties in medical 45
Practitioners, hints to young 341
Puberty, remarkable inftance of early 243
Publications, lifts of new medical 88, 176
Pyrolignite of iron, ufes of 439

Rabies canina, on the 53, 111, 343
——, queftion on 260
Radix rhatanæ, recommendation of 67, 107
Rattle-fnake, fatal effects of the bite of 525
Recovery, fingular cafe of fpontaneous 237
Rectum, on the fchirrhous contracted 281
Reeve, Dr. on cretinifm 9
Reform, refolutions refpecting a medical 328
——, obfervations on the plan of 465
Reft in phthifis, on 89
Reviewing, remarks on 441
Reynolds, on the cafe of Sir Jofhua 425
Rheumatifm, on the treatment of 348
Rhododendron ponticum, fugar exuded from the flowers of 261
Roberton's, Dr. remarks on ftricture 17, 126, 257, 346
—— reports of difeafes 84, 169, 257, 346, 433, 522
—— on hydrocephalus internus 378
Robertfon's, Mr. cafe of enlargement of the knee joint 515
Royfton's, Mr. fketch of the progrefs of medicine 1, 105
—— on mortality from phthifis 239
Rum, cafe of death after pumping of 29

Sagapenum, defcription of the gum 34
Saunders, Dr. on the hepatitis of India 415
Scarification, obfervations on 388
Scirrhous tumours, effects of a peculiar regimen on 79
—— parotid gland, on removing a 221
—— contracted rectum, on the 281
Scott's, Mr. cafe of death after pumping of rum 29
—— obfervations on fpider's web 94, 370
—— on the difeafes of feamen 373
Scrofula, obfervations on 494
Scrotum, cafe of froft-bitten 220
——, on the influence of cold applied to the 327
Seamen, on the difeafes of aged 373
Secretions, on animal 174
Serny, Dr. on difeafes of the eye 167
Sheep, on the inoculation of 87
Simmons's, Mr. cafe of purulent ophthalmia 252
—— apology for the cutting gorget 339
—— cafe of ague 467
—— on ophthalmia ib.
Small-pox infection, two cafes of 242
——, prevalence of 524
Smith, Mr. on the cutting gorget 224
——, Dr. on the fmall-pox 343
Spider's web, on the virtues of 94, 218, 369, 370
Spinal marrow, effect of upas on the 96
Spirit, apparatus for determining the quantity of 351
Spleen, obfervations on the 6, 39
Stanger's, Dr. cafe of obftinate cough 70
Strangulated fcrotal hernia, effects of cold on 233
Stricture, remarks on 17, 126
—— of the inteftine, cafe of 392
Stomach, on the functions of the 5
Stow, a chalybeate found near 346
Sugar exuded from the rhododendron ponticum 261
—— of lead, ufes of 350
——, fubftitutes for 525
Sulphuric ether, improved procefs of preparing 174, 362

Superfœtation, cafe of 47
———, obfervations on 293
Surgeons, proceedings of the royal college of 260
———, on the duties of navy 374
Syphilitic and gonorrhœal virus, identity of 391
Tacamahac, defcription of the 35
Taylor's, Mr. anfwer to Dr. Moffman 124
Tetanus, obfervations and cafes of 115, 324, 396, 479
———, cafes of 120, 185
Thomas's, Mr. cafes of artificial dilatation of the urethra 155
Thomas's, St. Hofpital, lectures at 262
Throats, on contagious fore 176
Tide, an extraordinary 527
Topography, on a medical 106
Tourniquet, on its application in intermittents 549
Tumour of the brain, cafe of 161
Tumours, effects of a peculiar regimen in fcirrhous 79
Tympanum, on perforating the 191

Ulcers, on the treatment of cancerous 79

Upas, its effects on the fpinal marrow 96

Urethra, on ftrictures of the 17, 126

———, artificial dilatation of the female 155

———, defcription of two mufcles furrounding the membranous part of the 159

———, cafe of hæmorrhage from the 341

———, Mr. Luxmoore on ftrictures of the 517

Vaccination, teft of fecurity for 173
Valentin's, Dr. remarks on hydrophobia 53
Variola and vaccina, coincidence of 87
Varnifh, a fine one obtained from copal 351
Vauquelin's, M. defcription of ca'aguala 87
Vegetables, on the action of 98
Virus, on fyphilitic and gonorrhœal 391
———, on the venereal 312
Vifion, obfervations on 307
Voltaic apparatus, new experiments with the 174

Wallajahad, mortality among the troops at 428
Walker, Dr. on vifion 307
Water in cancer, effects of 79
———, fimple method of filtering 439
Water-fpouts, account of 526, 527
Watts's, Mr. treatment of the diabetes, on 110, 252
White's, Mr. cafe of early puberty 243
——— obfervations on fcirrhous contracted rectum 281
——— cafe of cancer 355
Willan, Dr. on cutaneous difeafes 109
Wilfon's, Mr. defcription of two mufcles 159
———, Dr. account of experiments 253, 330
Wine in fever, on 41
Wollafton's, Dr. Galvanic experiments 174
Woolcombe, Dr. on confumption 239

Yeaft, obfervations on 403

Yellowly's, Dr. cafe of tumour in the brain 161

——— cafe of death produced by arfenic 427

Young's, Mr. cafe of a fœtus in the abdomen of a boy 166

Zingiberic, a new acid obtained from ginger 349

